# Model-based Traction Force Microscopy Reveals Differential Tension in Cellular Actin Bundles

**DOI:** 10.1371/journal.pcbi.1004076

**Published:** 2015-03-06

**Authors:** Jérôme R. D. Soiné, Christoph A. Brand, Jonathan Stricker, Patrick W. Oakes, Margaret L. Gardel, Ulrich S. Schwarz

**Affiliations:** 1 Institute for Theoretical Physics and BioQuant, Heidelberg University, Heidelberg, Germany; 2 Institute for Biophysical Dynamics, Department of Physics, and The James Franck Institute, University of Chicago, Chicago, United States of America; Cambridge University, UNITED KINGDOM

## Abstract

Adherent cells use forces at the cell-substrate interface to sense and respond to the physical properties of their environment. These cell forces can be measured with traction force microscopy which inverts the equations of elasticity theory to calculate them from the deformations of soft polymer substrates. We introduce a new type of traction force microscopy that in contrast to traditional methods uses additional image data for cytoskeleton and adhesion structures and a biophysical model to improve the robustness of the inverse procedure and abolishes the need for regularization. We use this method to demonstrate that ventral stress fibers of U2OS-cells are typically under higher mechanical tension than dorsal stress fibers or transverse arcs.

## Author Summary

Adherent cells respond very sensitively not only to biochemical, but also to physical properties of their environment. For example, it has been shown that stem cell differentiation can be guided by substrate rigidity, which is sensed by cells by actively pulling on their environment with actomyosin-generated forces. A commonly used method to measure cell forces during essential biological processes is traction force microscopy, which uses the deformations of a soft elastic substrate to calculate cell forces. However, the standard setup for traction force microscopy suffers from mathematical limitations in calculating forces from displacements. In order to improve this method, we combine image data and biophysical modelling to arrive at a procedure which is more robust and in addition allows us to make statements about the force distribution not only at the cell-substrate interface, but also inside the cell. Here we demonstrate this approach for the contractility of actin stress fibers, which we investigate experimentally with U2OS-cells and theoretically with an active cable network model.

## Introduction

**Fig 1:**
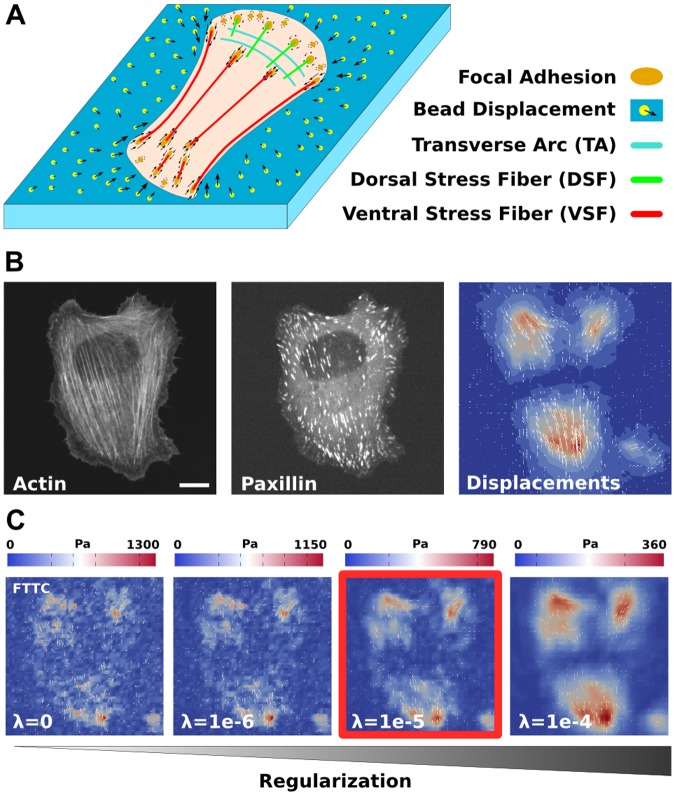
Actin cytoskeleton and traction force microscopy. (A) Schematics of a cell cultured on a soft elastic substrate with embedded fluorescent marker beads. Three different kinds of stress fibers and the actin network result in forces being transmitted to the substrate through focal adhesions. (B) Experimental data for a representative U2OS-cell. Actin and paxillin images show stress fibers and focal adhesions, respectively. Displacement data is extracted form the movement of the marker beads. Scale bar 10 microns. (C) Reconstruction of the traction forces with regularized Fourier Transform Traction Cytometry depends on the choice of a regularization parameter. The standard choice based on a Bayesian estimate is marked by the red box.

Adherent cells continuously probe the mechanical properties of their environment by exerting forces through integrin-based sites of adhesions (focal adhesions, FAs) [1,2]. These cellular forces are mainly generated by myosin II motors that interact with different types of actin networks and bundles [3,4]. The most prominent actin structures in cells cultured on flat surfaces are stress fibers (SFs), which have been further classified into different subclasses (Fig 1A) [5,6]. Transverse arcs (TAs) run parallel to the cell periphery and are connected to FAs only indirectly through dorsal stress fibers (DSFs), which emanate radially from peripheral FAs and run parallel to the dorsal membrane. Ventral stress fibers (VSFs) are connected at both ends to FAs and run parallel to the ventral membrane. Additionally the actin cortex and distributed actin networks contribute to force generation due to myosin II activity and actin polymerization. Together, the system built of FAs, SFs and actin networks regulates cell shape and the distribution of stresses on the substrate, thereby mediating the mechanical interactions of the cell with the extracellular environment [1,3,7]. Thus it is essential to develop methods to measure cellular forces and to associate them with individual components of this system in order to understand how cells precisely control force generation.

Different experimental methods have been developed before to measure cellular forces and to relate them to the structural organization of the cell. Forces at FAs have been measured with traction force microscopy (TFM) on soft elastic substrates [8–10], pillar arrays [11,12], and fluorescent force sensors [13–18]. TFM is the most direct and convenient method because it requires only small changes to standard cell culture protocols. Cells are plated on a soft polymer film (usually a polyacrylamide gel) with embedded marker beads (Fig 1A). An appropriate cell type for the study of stress fibers are U2OS-cells [6], which typically show a well-developed system of SFs and FAs (Fig 1B, S1). Taking a reference image with the cell removed from the substrate, the relaxation of the fluorescent marker beads can be used to extract substrate deformations (vector field in Fig 1B, combined with a contour plot for the magnitude). From this information the traction force field can be reconstructed (Fig 1C) and correlated with the internal actin structure, including actin retrograde flow and SFs [19,20]. In particular, it has been found that mature (μm-sized) FAs are often connected to stress fibers, which act as growth templates and force transducers at the same time, and transmit a typical force of a few nN [21,22].

Measuring forces inside the cell is much more challenging than measuring them at the cell-substrate interface. The contractile tension of a single SF can be estimated by laser cutting of individual stress fibers [20,23–25]. However, no experimental technique has been developed yet to measure forces inside the whole set of SFs in a cell. The mean force contribution of the actin cortex and distributed networks can be assessed by fitting whole cell contraction models to traction maps reconstructed in TFM experiments as shown first for small cell colonies [26–28] and recently also for single cells [29,30]. These measurements have revealed that the effective cortical tension is of the order of nN/μm [26,31].

From a mathematical point of view, the reconstruction of traction forces from bead displacements is an inverse problem of elasticity theory. Due to experimental noise in the displacement data, it is ill-posed and requires additional information to lead to a unique force estimate. To resolve this issue, one typically complements the reconstruction by a regularization scheme. This process amounts to making a priori assumptions on the expected force scale and the spatial distribution of cellular traction [8,32,33]. Typically, high local tractions and steep traction gradients are repressed, and the repression is adjusted by regularization parameters. This strategy has been shown to effectively reduce the effects of experimental uncertainties and to achieve unique solutions which however are influenced by the regularization procedure. A rigorous way to determine the optimal regularization parameter is missing. Usually one attempts to achieve optimal noise reduction by using the so-called L-curve criterion [34] or by force scale estimations using Bayesian theory [33,35]. The choice of the regularization parameter has a strong influence on the reconstructed traction distribution, see Fig 1C (the solution marked in red is the standard choice based on a Bayesian argument). If it is chosen too small, the calculated forces are dominated by noise rather than by cellular processes. If it is chosen too large, the details of the force field are smoothed out and the overall force magnitude is too small. Moreover the noise conditions, and thereby the optimal parameter values, may also vary within a set of experiments.

In order to improve the standard procedures in traction force microscopy, here we introduce model-based traction force microscopy (MBTFM). The main idea is to complement the traction reconstruction process with more information beyond the displacement data, including a biophysical model that represents the essential features of interest. Therefore the model choice can be variable and might be different when e.g. studying migration of rapidly moving cells versus mature adhesion of immobile or slowly migrating cells. In any case, image processing can be used to extract prominent features of the cell type under investigation and to augment the biophysical model with this information. Here we implement this approach for U2OS-cells that show mature adhesions and whose contractile machinery is strongly dominated by actin SFs. Image processing is used to segment FAs and SFs, and this information is utilized for building a cell model. By supplementing TFM with a specific whole cell model, one creates a better defined inverse procedure based on biophysical arguments that allows one to improve standard regularization techniques. In particular, an appropriate choice of the biophysical model might allow one to abolish the need for regularization because the model might be sufficiently constraining to exclude unreasonable solutions. Once established, the cell model can be interrogated for biologically relevant questions. In the context of the maturely adhering U2OS-cell studied here, we ask how force is distributed statistically over the ensemble of different SFs and how much actin network contraction contributes to the overall amount of cell tension.

In this study we chose an active cable network as a minimal model to represent the two main features of actomyosin contractility in U2OS-cells, namely the constant pull by the myosin II motors and the asymmetry between stretching and compression dominating the mechanical response of an actin bundle [36] (S2 Fig). We give a detailed description of the method and demonstrate the application to experimental data. In particular we show that for U2OS-cells, actin SFs represent the main source of cellular traction forces while contraction of actin networks have a minor influence. Further we found that ventral SFs are significantly more contractile than dorsal SFs and transverse arcs, in agreement with earlier work on the molecular composition of the different structures.

## Methods

### Cell culture

U2OS human osteosarcoma cells (ATCC) were cultured in McCoy's 5A media (Sigma) supplemented with 10% fetal bovine serum (HyClone), 2 mM L-glutamine (Gibco) and penicillin-streptomycin (Gibco). U2OS-cells were transfected with plasmid DNA constructs encoding for GFP-actin (gift of the Gary Borisy Lab, Northwestern University) and mApple-paxillin using the transfection reagent FuGENE 6 (Roche). Cells were plated and allowed to attach and spread for approximately 16 hours prior to imaging.

### Polyacrylamide substrates for traction force microscopy

Polyacrylamide (PAA) substrates containing far-red fluorescent microbeads (Invitrogen, d = 40 nm) were prepared on glass coverslips using previously published methods^10,37^. Briefly, PAA gels with 7.5%/0.1% weight percentage of acrylamide/bis-acrylamide was used to create a gel with a shear elastic moduli of 8.4 kPa [37,38]. Fibronectin (Millipore) was coupled to the surface of the PAA gels by means of hydrazine hydrate [22,37,39] as previously described. PAA gels were incubated for at least 2 hours in undiluted hydrazine, followed by a 1 hour incubation in 5% acetic acid and then washed. A 10 μg/mL fibronectin solution was prepared in sodium acetate buffer (pH 4.5), and oxidized by addition of 40 μg/mL sodium meta-periodate prior to a 30 min incubation on the PAA gel at room temperature. The PAA gels were then rinsed repeatedly and plated with cells.

### Microscopy

Live cell traction force measurements were performed on an inverted Nikon Ti-E microscope with a CSU-X confocal scanhead (Yokogawa), laser merge module containing 491, 561 and 642 nm laser lines (Spectral Applied Research) and an HQ2 cooled CCD camera (Roper Scientific). All hardware was controlled via Metamorph acquisition software (MDS Analytical Technologies). Traction force data was obtained at 37°C in a perfusion chamber (Warner Instruments) using a 60x 1.2 NA Plan Apo WI objective (Nikon). Cells were maintained in culture media supplemented with 10 mM HEPES and 30 μl/ml Oxyrase (Oxyrase, Inc.).

### Displacement analysis and FTTC force reconstruction

Methods for traction force microscopy have been previously described [10,22,37]. Briefly, images of fluorescent beads embedded in the PAA gel were aligned to compensate for experimental drift and the bead displacement field was calculated between pairs of images by comparing the unstrained bead images obtained after the cell had been removed to images obtained with an attached cell. Displacement fields were calculated using Particle Imaging Velocimetry (PIV) software in MATLAB (available at http://www.oceanwave.jp/softwares/mpiv/), using the Minimum Quadratic Differences (MQD) algorithm which calculates the shift necessary to produce the minimum cross-correlation coefficient between a small region of the experiment image and the reference image. Displacement vectors were filtered and interpolated using the kriging interpolation method. We used a displacement grid size of 0.86 μm for these measurements. From the displacement data, Fourier transform traction cytometry (FTTC) [9] was then used to estimate traction stress [10]. Traction stresses were reconstructed with zeroth-order regularization, which has been shown to yield traction force measurements consistent with the boundary element method [10].

### Model choice

In this study we correlate cellular forces with SFs and FAs. For this purpose we need to utilize a mechanical model for the entire cell that allows us to describe its force generating and transmitting behavior. Several models describing forces of adherent cells have been developed over the past decade. Contour models have been shown to give reasonable estimates for cell forces and shapes if there are no prominent internal structures [29,36]. Continuum mechanics models focus on the elastic properties of the bulk cell material [40–42]. In a recent study, these approaches have been combined in a continuum mechanics model with line tension [30]. However, all of these models have a continuum character and none of them can easily implement differential tensions in discrete stress fibers. For pillar assays, truss models have been used to estimate tension in internal stress fibers from post displacements [43,44], but these models did not consider the effect of the cell body and work only for a small number of adhesion sites.

For our model choice, we were guided by the following four principles. (1) U2OS-cells are a cell type characterized by prominent stress fibers that have to be modeled as discrete elements. (2) Stress fibers are under tension and the tension may vary between individual SFs. (3) There is a homogeneous contractile tension in the cell resulting from various distributed actomyosin networks not visible with the standard optical microscope. (4) Forces are transmitted to the extracellular space mainly via focal adhesions. A suitable framework to implement these assumptions is a network of active cables [45]. A cable responds like a Hookean spring to extension while it does not resist compression, and an active cable additionally features a constant contractile tension. Each link of the network is therefore associated with the energy E_i_ = l_i_ T_i_+k (l_i_-l_i,0_)^2^/2 for l_i_>l_i,0_ and E_i_ = l_i_ T_i_ for l_i_≤l_i,0_, where l_i_, l_i,0_ represent the actual and the rest length of link i, respectively, T_i_ is its active tension, and k is the spring constant for the elastic regime. This model represents several typical mechanical properties of the actin cytoskeleton. If under compressive load, filaments can slide telescopically along each other, or depolymerize or buckle, while they respond elastically to stretch [46]. Cell area is not conserved because the model only considers the two-dimensional projection onto the substrate, thus cellular material can be exchanged with the third dimension. The constant contractile tension arises from myosin II motors that work in the stall regime. Active cable models have been shown to correctly predict shapes of adherent cells on micro-patterned substrates and yield force distributions that are robust with respect to local changes in network geometry or topography [45]. Note that this differs remarkably from networks of Hookean springs. Springs in particular propagate compressive force modes over long distances, which do not appear in cables by definition. In order to achieve a close relation to experiments, the model is built directly from image data.

### Image processing and model generation

**Fig 2:**
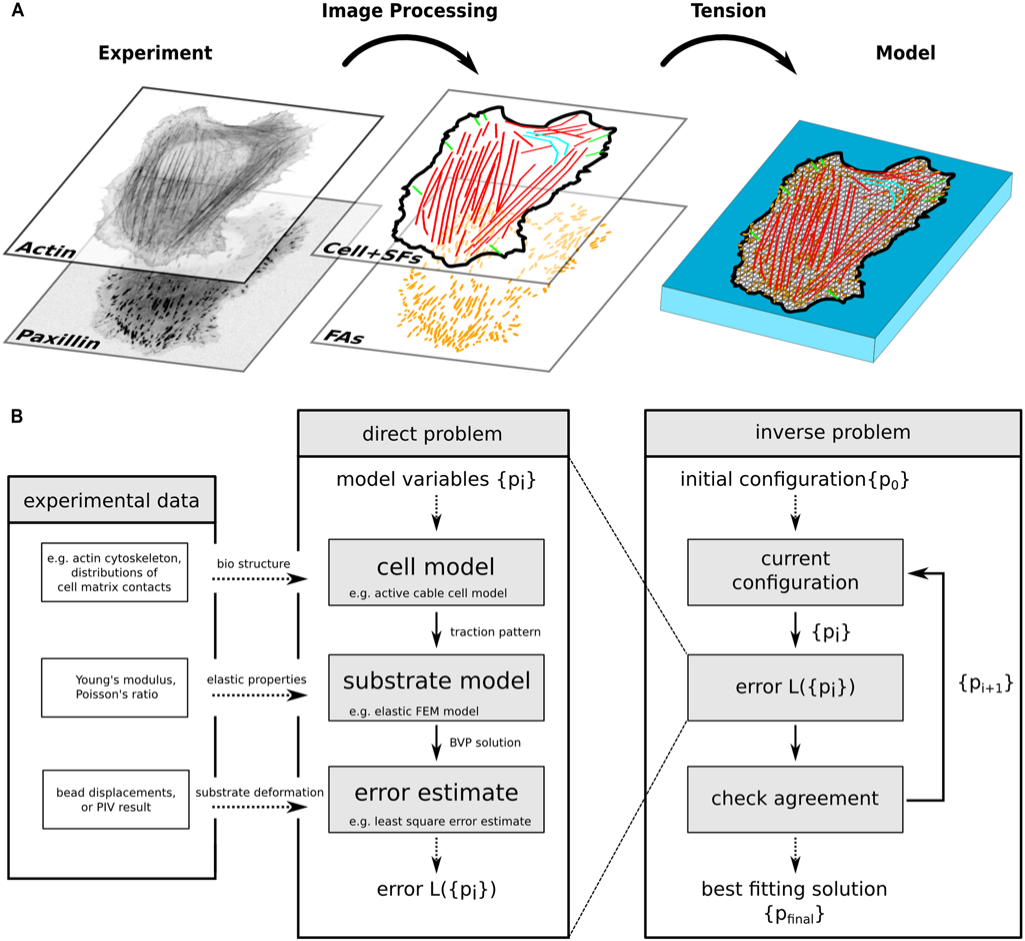
Computational workflow of MBTFM. (A) Actin and paxillin images are segmented and converted into a whole cell model, with an individual tension value assigned to each stress fiber and one global tension value assigned to the actin networks of the cell. (B) Each set of model parameters leads to an error estimate which is then minimized to estimate the best fit to the experimentally measured displacement field. In contrast to standard traction force microscopy, no regularization scheme is required for MBTFM.

To generate the biophysical model for a specific cell, we wrote a new plugin SoFAST (Segmentation of Focal Adhesions and Stress Fibers) for the image processing suite ImageJ [47] and proceed as follows (Fig 2A). First we segment FAs and stress fibers from paxillin and actin fluorescence images, respectively. Here it is important to avoid undersegmentation (cf. results section). Second, we classify SFs following the definitions of Hotulainen et al. [6], where we also utilize information about FA locations. Third, a mechanical network of nodes and links is fitted into the cell shape as segmented from the actin image. SFs are embedded into the network as lines, irrespective of their type. While these lines are fixed, we use the Distmesh algorithm [48] to achieve a homogeneous mesh size. We then fix nodes in the proximity of FAs.

Now the network can be contracted by assigning a global tension to the network and one tension value to each individual SF, resulting in less than 100 degrees of freedom (compared to many thousands of degrees of freedom in traditional TFM). The resulting force at each fixed node is calculated and mapped to the closest FA. Note that there can be more than one node fixed for a single FA, depending on its area. Finally, cellular traction spots are defined by the shape and area of an ellipse fitted to the FA, and the force vector sum of all fixed nodes mapped to the particular FA.

### Model for the soft elastic substrate

Displacement fields can be obtained from cellular tractions with a material model of the soft elastic substrate. Substrates used in our experiments are isotropic with a Young's modulus of several kPa. Since cellular traction stresses are on the order of several hundred Pa, deformations are small (few μm). Moreover the deformation gradients are always smaller than 1 (maximal value 0.15). Therefore substrate deformations can be calculated in the framework of linear elasticity theory. Several analytic solutions exist for specifically shaped traction spots on the surface of an elastic half space, e. g. for circular spots or the Boussinesq solution for point forces [49]. In order to be able to also treat FAs with non-circular (elliptical) shape, here we use a finite element method (FEM) approach.

The elastic problem is stated as a boundary value problem (BVP), where cellular traction stress defines the boundary condition at the substrate’s top surface. The bottom surface is rigidly fixed to a glass cover slip, and therefore all displacements have to vanish there. The free boundaries at the sides are assumed to be stress free. To ensure that the latter is a justified assumption, the substrate model is extended in lateral direction on each side by 30 μm (compared to the visible region of the image data, which is ca. 100x100 μm^2^). Our software uses the public domain FEM-library deal.II [9]. A three-dimensional substrate mesh is generated using built-in functions. In order to save computation time, but still keep a high local resolution, local mesh refinement is applied to the top surface. Starting from a uniform rectangular mesh, elements containing fixed vertices from the active cable network are split in half both horizontally and vertically. This is repeated until a desired mesh size around FAs is achieved.

### Optimization

With the cell and substrate models described above, we are now able to calculate a simulated displacement field for a given set of model parameters. The intention of MBTFM is, however, to solve the inverse problem of finding the optimal set of model parameters (and thereby the reconstructed tractions) for a given cellular displacement field (Fig 2B). To define optimality, we need to specify an error estimate for the deviation of the experimentally measured field and one that is simulated for a given set of model parameters. This is achieved by the least squares estimator L_2_ = (1/N) ∑_x_ (d_e,x_-d_s,x_)^2^ (L_2_-norm), where the sum runs over all locations of displacement measurements for the discrete experimental displacement field, N is the number of data points, and d_e_, d_s_ are the experimental and simulated displacements. We can then utilize standard optimization techniques to find an optimal solution.

For the active cable network, we apply an adapted version of the conjugated gradients algorithm. Since the change in tension in a single stress fiber affects the traction distribution more locally than an alteration in the global background tension, we separate the optimization into blocks. We perform repeated optimizations of the fiber tensions, before we adjust the background tension. This prevents the algorithm from stalling and leads to reasonable convergence at ~ 200 optimization steps. Note that every minimization iteration involves several calculations of both cellular network contraction and FEM substrate deformation. As this is expensive in terms of computation time, the numerical work is parallelized using the boost thread library [50]. In this way, multiple points of the L_2_-landscape with varying stress fiber tensions can be explored at the same time, leading to faster calculations of the high-dimensional gradients and quicker line minimizations. The computation time on 8 cores of current Intel i7 processors is on the order of 15 minutes per iteration step.

For later analysis, we define two instructive measures for whole cell contractility obtained by our optimization approach. After contraction with the optimal set of stress fiber tensions and the additional background tension, the sum of absolute resulting forces at all focal adhesions is called the total force. The network force on the other hand is determined by setting all stress fiber tensions to the background tension.

## Results

### MBTFM workflow

We started by segmenting fluorescent images for SFs and FAs and constructing a cell-specific active cable model that spans the whole cell (Fig 2, S2 Fig). We combined this model with a FEM-representation of the soft elastic substrate in order to be able to directly compare predicted and measured displacements. Starting from all tensions set to zero, we optimized for the model parameter set with the best agreement of the two displacement fields. Although not directly part of the optimization process, traction forces then could be directly inferred from the active cable network.

### Distribution of focal adhesions and stress fibers

**Fig 3:**
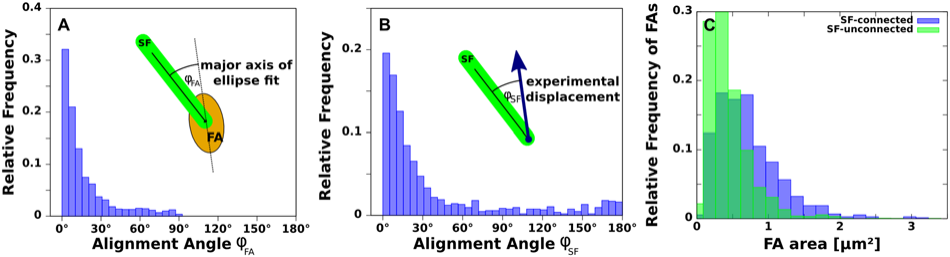
Orientation analysis of focal adhesions, stress fibers, and local displacements for U2OS-cells. (A) Relative angular distribution of FAs and attached SFs (top, n = 1305). (B) Relative angular distribution of local displacements at anchoring points of SFs (middle, n = 1297). (C) Area distribution of mature FAs with (blue) and without attached SFs (green) (bottom,n = 3612). The distributions are based on a data set of 16 U2OS-cells on soft elastic substrates (Young's modulus E = 8.4kPa).

In order to study the influence of actin SFs to the measured substrate deformations and their correlation with FA orientation and shape, we analyzed the alignment of FAs, SFs, and local displacement directions at anchoring points of SFs to the substrate. For this task we segmented all SFs and FAs from a data set of 16 U2OS-cells. We fitted an ellipse to each segmented FA and evaluated the direction of the main axis and the corresponding area. We found that FAs connected to a SF are highly aligned with them (Fig 3A). Further we could also observe an alignment of SF direction and local substrate displacement at the anchoring points (SF end attached to a FA), see Fig 3B. These alignment distributions become even more peaked when given a stronger weight to larger deformations. The derived angular distributions indicates a strong influence of actin stress fibers on both the maturation of FA and the force transmission to the extracellular environment. Additionally we found that the size distributions of FAs with and without attached SF significantly differ (Fig 3C). The possibility of a FA to be larger than 1μm is considerably larger for FAs with attached SF than for FAs without. Together these results support the major model assumptions used by the actin cable network cell model applied in our MBTFM framework.

### Robustness of the method

**Fig 4:**
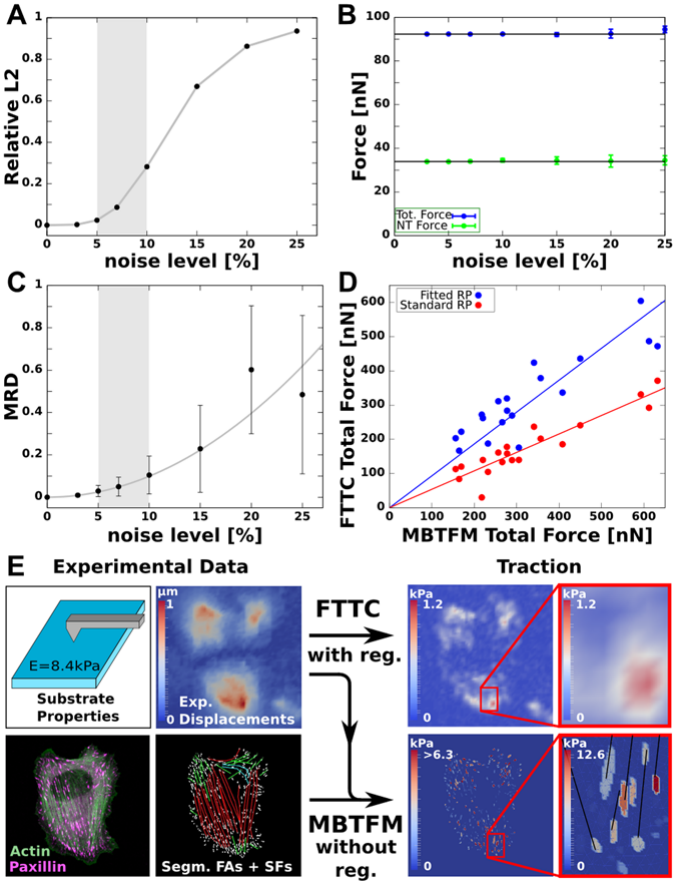
Robustness of MBTFM and comparison with FTTC. (A) Realistic traction patterns are generated by calculating the direct problem for a known test tension distribution. Gaussian noise is added to the resulting displacement vectors. The noise level is defined with respect to the largest displacement in the whole field. With increasing noise level the L_2_ error estimate increases continuously as expected. (B) Total forces and network forces reconstructed with MBTFM are not affected by the noise level in the simulations, in marked contrast to standard reconstruction methods like FTTC. (C) The precision of tension predictions for individual stress fibers decreases for higher noise level (MRD: mean relative deviation). By evaluating experimental displacement data for noise in traction-free regions, we find a typical experimental noise level between 5–10%. In this region (gray), the MRD does not exceed 10%, which we thus identify with the accuracy of our tension reconstruction for stress fibers. (D) Direct comparison of the total force obtained with FTTC and MBTFM reveals a linear relationship (red). The slope of the linear fit line here depends on the regularization parameter alone. By fitting the regularization parameter to a one-to-one relationship (blue), FTTC can be calibrated based on the biophysical model input instead of traditional noise optimization (red). (E) Comparison of the standard TFM method FTTC and MBTFM. Based on the additional experimental data, the model can achieve a more detailed traction map. Further it allows us to directly map tensions in single stress fibers (black lines in inset) to experimental displacements.

Because MBTFM does not include a regularization scheme, we investigated how it performs in the presence of noise. The spatial resolution of TFM is mainly constrained by experimental uncertainties in measuring bead displacements, which originate from limited optical resolution of the microscopy setup, uncertainties in the image processing procedures and heterogeneities in the substrate material with its embedded marker beads. The uncertainty in a given data set can be determined by analyzing the distribution of absolute displacement magnitudes at cell-free regions of the substrate image. Such evaluations led to Gaussian-shaped distributions in our data set, as reported earlier [33]. We therefore summarize the possible uncertainties under the term *displacement noise*, for which we find a typical value of 5–10%. In order to test the performance of MBTFM in an experimental context, we first simulated its ability to reconstruct a given traction pattern in the presence of such displacement noise (Fig 4A-C). The deviation between the theoretical prediction and experimental measurement is represented by the relative L_2_-norm that ranges between 0 for perfect agreement and 1 for a vanishing force field. We sampled 10 different displacement fields for each noise level and averaged over the reconstruction results. While the L_2_-norm naturally approaches 1 for higher noise levels (Fig 4A), we find that MBTFM still performs very well in the experimentally relevant range of displacement noise from 5–10% (Fig 4B+C). Interestingly, the reconstructed total force remains almost constant over the entire range of simulated noise levels, which confirms the robustness of the method (Fig 4B). In a second evaluation of simulated data, we checked the influence of erroneous segmentation (S3 Fig). We find that segmenting too few SFs leads to a force shifting to neighboring fibers. Because cable networks do not propagate compression, this remains a local effect [45], which is also verified in our test reconstructions. On the other hand, segmenting too many stress fibers barely affects the reconstruction result as these additional degrees of freedom do not have to be used by the optimal solution. We conclude that it is important to avoid undersegmentation rather than oversegmentation of SFs and that MBTFM performs very well in the presence of displacement noise despite the fact that it does not use any regularization scheme. This shows that our biophysical model is a reasonable assumption that leads to well-defined solutions.

In general, we cannot rigorously prove that the results of our method are unique. However, we performed several types of simulations to demonstrate that under realistic conditions our reconstruction method comes close to the global minimum. First, we used different initial tension configurations for the reconstruction of simulated data. They all converged to the same correct L_2_ minimum for the examined test cell geometries. If there were many local minima other than the correct minimum in the L_2_-landscape, one would expect the optimization to stall in a local minimum for some initial configuration instead. Second, when we explored the effect of displacement noise, we observed that the final stress fiber configuration deviated only slightly from the correct solution for small noise levels. This also points to the existence and identification of a single global minimum, albeit this is slightly shifted due to non-vanishing noise.

### Comparison with FTTC

One of the most common techniques to reconstruct traction force based on TFM data is Fourier Transform Traction Cytometry (FTTC) [9,10]. In addition to the substrate-related information that is used in FTTC, MBTFM also uses fluorescence image data of the cell and a biophysical model (Fig 4E). Together this enables MBTFM to dispense with regularization. In order to quantitatively validate and compare our method with this well-established approach, we systematically analyzed experimental data using both methods. We find that the resulting total forces are linearly correlated between MBTFM and FTTC reconstructions (red symbols in Fig 4D). In contrast to MBTFM the results of FTTC depend on a regularization parameter and are systematically smaller when based on optimal noise reduction (red line has a slope smaller than 1). By fitting the regularization parameter, we can achieve good agreement between the two methods (blue symbols, now the blue line has slope 1). From this we conclude that MBTFM represents an alternative way to calibrate the regularization scheme independent of standard optimal noise reduction [34] or force scale arguments [33] and based on biophysical model considerations alone.

### Estimating tensions in individual SFs

**Fig 5:**
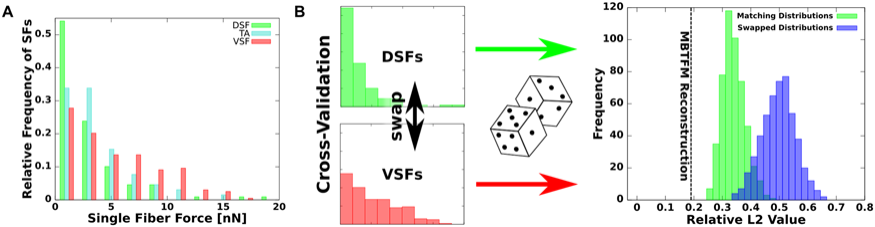
Statistical distribution of stress fiber tensions in U2OS-cells. (A) Histogram of single stress fiber tension values sorted by fiber types (16 U2OS-cells, N = 369 SFs). All three segmented types show a broad distribution due to biological variability, but VSFs are on average under the highest tension. (B) The statistical significance of the different distributions is verified by performing a consistency check in which the distributions are scrambled. We swap the distributions for the tensions of dorsal and ventral SFs (the tension distribution for transverse arcs is left unchanged) and then assign new tension values for a specific cell by drawing from these distributions. Because the new tension values are generated by random, none of them is as good as the optimal set (dashed line). However, repeated simulation with matching distributions (green) lead to significantly better error estimate values than using a swap of DSF and VSF distributions (blue).

A further substantial feature of MBTFM is its capability to directly associate measured substrate deformation with a certain configuration of intracellular tension. Note that the displacements and not tractions constitute the experimental data in TFM. As our model yields contractile forces for all SFs in the cell, we can assess their statistical distribution. We have analyzed a data set of 16 U2OS-cell with 369 segmented SFs in total. We found that the distributed actin networks never carry more than 20% of the overall force. We further derived a statistical distribution of non-vanishing tension values over the different types of SFs (Fig 5A). As a result we found a broad statistical distribution of tension among the various stress fiber types, with VSFs being the most likely type of stress fiber to be under high levels of tension, followed by TAs and DSFs. These findings agree with the experimental observation that VSFs typically show myosin II striation and pull against FAs at both ends, while DSFs do not contain myosin II [6,51]. TAs seem to adopt an intermediate state with myosin II striation but no clear attachment to FAs. In order to demonstrate the significance of the extracted distributions, we performed the following consistency check. One cell was selected and new tension values were attributed to each fiber by drawing from statistical distributions. When drawing from the extracted distributions (green), we found a L_2_-norm that is close to the optimal value (result from the minimization procedure, dashed line). When drawing from scrambled distributions (ventral and dorsal SF distributions swapped, TA distribution unchanged, blue), the L_2_-norm is clearly much worse. This shows that the details of the distributions matters, despite the fact that they are relatively broad, and that ventral SFs are indeed the strongest types of contractile elements.

## Discussion

Here we have introduced a novel method to reconstruct cellular forces from the deformation of elastic substrates. The main idea of model-based traction force microscopy (MBTFM) is to complement the traction reconstruction process by a whole cell mechanics model based on additional image data. The proposed concept is very general in the sense that several whole cell models can find application in MBTFM, depending on the cell type and the scope of the study. In this article we focused the analysis on a cell contraction model based on active cables, which facilitates the analysis of actin stress fiber structures as they typically occur in U2OS-cells.

Compared to simpler models like contour models [29,31] or continuum mechanics models [30,40], our approach is particularly suited to analyze discrete elements of the actin cytoskeleton. Compared to earlier studies that used truss models to evaluate a few stress fiber tensions on pillar arrays [43,44], we have implemented this procedure for cells on flat elastic substrates with hundreds of FAs. In particular, we are able to estimate for the first time the intracellular distribution of contractile tension over a large ensemble of many different fibers. By using the active cable network model, we assumed that cellular forces are generated by contraction of the actin CSK and that actin networks behave mechanically like cables under application of contractile tension [45]. Another crucial assumption is that forces are exclusively applied via focal adhesions. Both assumptions seem reasonable for U2OS-cell. However, these are not applicable for arbitrary cell types and cell states. The main reason is a variety of force generation processes are known to take place in the cell besides myosin-based contractility, including actin polymerization and membrane blebbing. Moreover force might be transmitted also through other adhesion structures than mature FAs, e.g. through nascent adhesion in spreading and migrating cells [52–54] or through podosomes and invadopodia in a variety of cell types [55] (but not the U2OS-cells studied here). While we cannot exclude that small structures invisible under the fluorescence microscope transmit some of the traction forces of U2OS-cells, we observed that the occurrence of prominent FAs strongly coincides with high stress transmission as confirmed by traditional regularized TFM and our orientation analysis (Fig 3).

Compared with traditional TFM approaches like FTTC, MBTFM allows us to obtain unique results independent of any regularization parameter. In the context of TFM, the active cable network should not be interpreted as a detailed biophysical model of the cell, but rather as a reasonable reduction of parameter space that restricts the set of possible solutions based on biophysical relevance. This of course like traditional regularization biases the results towards a priori assumptions. However, the solution target is compared to traditional methods not motivated by simple noise reduction considerations but by a more detailed knowledge about the force generating biological system. Note that at the same time, MBTFM can be more robust to experimental noise than traditional TFM. We also showed an alternative way to calibrate standard reconstruction techniques by fitting the regularization parameter towards a best agreement with exemplary MBTFM results.

An additional advantage of the MBTFM approach is the inherent coupling of calculated substrate deformations with cellular mechanics. This allowed us to correlate the intracellular distribution of model tension with common TFM data. In this first application of the described method, we analyzed a data set of 16 U2OS-cells with N = 369 segmented SFs in total with the goal to investigate non-invasively the distribution of tension over the actin network and different types of SFs. As result of this evaluation we found that ventral SFs seem to be statistically stronger than other SF types. This is in good agreement with the distinct molecular structure of these fiber types as reported by various published studies [6,51]. We further successfully cross-checked the validity of this important result by simulations in which we scrambled the tension histograms.

MBTFM as introduced here strongly depends on very good image quality for building the biophysical model. Unfortunately, many common perturbations of the force-generating processes (e.g. pharmacological inhibitors like blebbistatin or latrunculin, or siRNA-knockdowns) lead to reduced contrast of the paxillin and actin images used here. In this case, our approach based on segmentation of prominent actin and adhesion structures can not be used and traditional TFM has to be preferred. Thus our approach is best suited to study cells with mature cytoskeletal and adhesion structures, like the wildtype U2OS-cells studied here.

In the future, our method can be used to dissect out the different mechanisms leading to the measured tensions, including myosin II contractility and retrograde flow. Because the method requires good contrast in the fluorescent data, one appropriate avenue for future progress seems to be the study of dynamical responses in wildtype cells, as they can be probed for example by laser cutting [20,23–25]. Using an appropriately adapted model, such an approach should allow us to dissect the details of stress fiber crosslinking in adherent cells. Another possible subject of large biological interest would be the extension to three-dimensional situations [56,57]. For a complete 3D-setup in the spirit of MBTFM, one had to track bead displacements, cell contour and internal cell structures in 3D, which is a large challenge to current microscopic techniques. If accomplished, however, such studies then will yield new mechanical insight into the mechanisms of global force transmission and sensing in the actin cytoskeleton of adherent cells.

## Supporting Information

S1 Fig Data for three representative U2OS-cells on soft elastic polyacrylamide substrates (E = 8.4 kPa). Cell 1 has also been used for Fig 1. For each cell, the following data is shown: raw images of actin and paxillin fluorescence, segmented stress fibers, segemented focal adhesions, generated active cable network (link length 1 micron) and resulting MBTFM traction reconstruction. Stress fiber color code: dorsal stress fibers (green), transverse arcs (turquoise) and ventral stress fibers (red). Red dots in the network indicate locations of focal adhesions, which are treated as fixed points during model network contraction. Scale bar 10 microns.
(EPS)

S2 Fig Active cable networks. (Top) Force-extension curve for a single active cable. In the extension regime, L>L_0_, the cable responds linearly elastic, i.e. like a Hookean spring. In the compression regime, L<L_0_, the cable does not resist deformation and the curve is flat. In addition, the cable is assumed to experience an active contractile tension, T, which shifts the passive force-extension curve up by a fixed amount. This tension is assumed to break down if the length drops below a critical length L_C_ = 0.01 L_0_). (Bottom) Snapshot of a representative active cable cell model. The mesh was generated with the SOFAST ImageJ-plugin and contains stress fibers (color-coded for type) and fixed points at locations of focal adhesions (red dots). The network link length is approximately 1 micron. The boxed region is shown as an inset on the right.
(TIFF)

S3 Fig Effect of segmentation procedures. In order to explore the effect of our segmentation procedures on the force predictions, we considered three typical situations. (A) As an example for oversegmentation, we introduced three arbitrary additional VSFs (yellow arrowheads). Scale bar 10 microns. (B) As an example of undersegmentation, we removed two DSFs (yellow arrowheads). (C) As another example of undersegmentation, we removed one VSF (yellow arrowhead). (D) L_2_-optimization with the conjugated gradient method as a function of the perturbations (A-C). The result is changed only little by the oversegmentation (A). In marked contrast, the two undersegmentations (B,C) lead to much worse results as shown by the large L_2_-norm. (E) Detailed analysis of the two undersegmentations. Stress fibers close to the missing ones (red arrowheads) try to compensate for the missing information and thus show very large deviations (more than 50%). (F) Effect on total and network force. Again oversegmentation does not change the result, while undersegmentation leads to a larger network force compensating for the reduced possibility to contract. The overall force nevertheless decreases because the anisotropic elements are missing. In summary, our procedure works well as long as the image data is not undersegmented.
(EPS)
