# Supplementary figures and images for: Model-based Traction Force Microscopy Reveals Differential Tension in Cellular Actin Bundles
Source: PLoS Comput Biol. 2015 Mar 6;11(3):e1004076. doi: 10.1371/journal.pcbi.1004076 (PMC4352062; doi:10.1371/journal.pcbi.1004076)

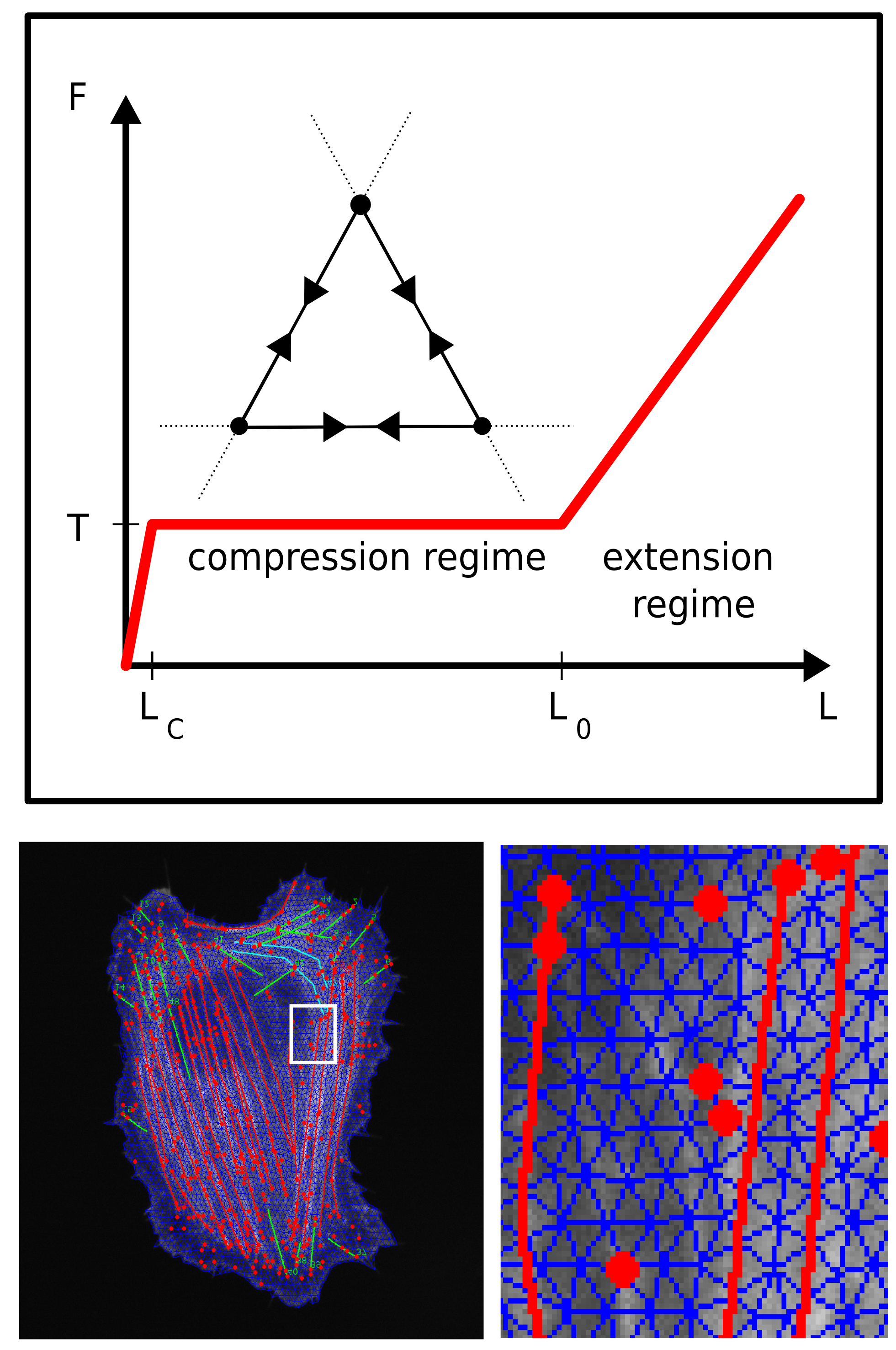

Supplement: S2 Fig — (Top) Force-extension curve for a single active cable. In the extension regime, L>L0, the cable responds linearly elastic, i.e. like a Hookean spring. In the compression regime, L<L0, the cable does not resist deformation and the curve is flat. In addition, the cable is assumed to experience an active contractile tension, T, which shifts the passive force-extension curve up by a fixed amount. This tension is assumed to break down if the length drops below a critical length LC = 0.01 L0). (Bottom) Snapshot of a representative active cable cell model. The mesh was generated with the SOFAST ImageJ-plugin and contains stress fibers (color-coded for type) and fixed points at locations of focal adhesions (red dots). The network link length is approximately 1 micron. The boxed region is shown as an inset on the right. (TIFF) [file pcbi.1004076.s002.tiff]
